# Comparative analysis of multi-zone peritumoral radiomics in breast cancer for predicting NAC response using ABVS-based deep learning models

**DOI:** 10.3389/fonc.2025.1586715

**Published:** 2025-05-14

**Authors:** Minfang Wang, Wanjun Chen, Ruiping Ren, Yuanwei Lin, Jiawen Tang, Meng Wu

**Affiliations:** ^1^ Department of Ultrasound, The Affiliated People’s Hospital of Ningbo University, Ningbo, Zhejiang, China; ^2^ Department of Radiotherapy and Chemotherapy, The Affiliated People’s Hospital of Ningbo University, Ningbo, Zhejiang, China; ^3^ Department of Radiology, The First Affiliated Hospital of Ningbo University, Ningbo, Zhejiang, China; ^4^ Department of Pathology, The Affiliated People’s Hospital of Ningbo University, Ningbo, Zhejiang, China

**Keywords:** breast cancer, neoadjuvant chemotherapy, radiomics, automated breast volume scanning, peritumoral features, artificial intelligence

## Abstract

**Background:**

Peritumoral characteristics demonstrate significant predictive value for neoadjuvant chemotherapy (NAC) response in breast cancer (BC) through tumor-stromal interactions. Radiomics analysis of peritumoral regions has shown robust capability in predicting treatment outcomes; however, the optimal peritumoral thickness for maximizing predictive accuracy remains undefined.

**Objective:**

To establish a clinically implementable framework for early identification of NAC non-responders through standardized prediction modeling. This study aims to determine the optimal peritumoral thickness for NAC response prediction by training and systematically comparing artificial intelligence (AI)-driven radiomics models across multiple peritumoral zones using Automated Breast Volume Scanning (ABVS).

**Methods:**

A total of 402 BC patients who received NAC were retrospectively analyzed. Pre-treatment ABVS images were processed to extract radiomic features from five regions of interest (ROIs): the intratumoral region (R0) and four consecutive peritumoral zones (R2-R8) extending outward at 2-mm intervals. The study cohort was divided into training and testing cohorts. ROI-specific TabNet models were developed using the training cohort data. Comparative analysis was performed in the testing cohort through comprehensive performance evaluation, including discrimination, calibration, clinical utility assessment, and classification metrics, to identify the optimal peritumoral zone. The radiomics features of the best-performing model were ranked by importance, with subsequent ablation studies validating the predictive contribution of high-ranking features.

**Results:**

Among the study population, 138 patients (34.3%) were classified as NAC non-responders. Model evaluation demonstrated progressively improved predictive performance from R0 to R6, with area under the ROC curves increasing from 0.681 to 0.845. The R6 model demonstrated optimal performance with accuracy of 0.810 and precision of 0.765. The combined model integrating R0 and R6 features enhanced predictive capability, achieving accuracy of 0.909, precision of 0.841, and recall of 0.902. Feature importance analysis identified textural heterogeneity and volumetric characteristics as the most influential variables, with the top features derived predominantly from the 6-mm peritumoral region.

**Conclusion:**

The 6-mm peritumoral zone demonstrated optimal predictive value for NAC response, with the AI-driven combined intratumoral-peritumoral model achieving superior performance. This standardized ABVS-based radiomics approach enables early identification of potential NAC non-responders, facilitating timely therapeutic modifications.

## Introduction

Peritumoral characteristics have emerged as crucial predictors of neoadjuvant chemotherapy (NAC) response in breast cancer (BC) (1, 2). The biological basis for this predictive capacity lies in the complex interactions within the tumor microenvironment, where stromal cells, immune infiltrates, and extracellular matrix components collectively influence therapeutic outcomes (3). Multiple studies have demonstrated significant correlations between peritumoral features and treatment responsiveness, suggesting that these surrounding regions contain valuable prognostic information beyond conventional intratumoral assessment (4–6).

The characterization of complex peritumoral features requires sophisticated computational analysis. Radiomics, as an advanced analytical approach, enables systematic extraction of imaging features through automated computational algorithms (7). It enables quantification of multiple high-dimensional features imperceptible to visual inspection, including complex mathematical descriptors of texture patterns, spatial relationships, and structural heterogeneity, thereby extending imaging phenotype characterization beyond conventional radiological assessment (8, 9). Growing evidence suggests that the optimal extent of peritumoral region represents a critical parameter in radiomics analysis. Initial investigations have examined various peritumoral zones (1mm to 10mm) for BC molecular subtyping and lymph node metastasis assessment (10–13). These studies have validated the contribution of optimized peritumoral extent to improved predictive modeling. Nevertheless, systematic evaluation of optimal peritumoral thickness for NAC response prediction remains inadequately explored.

Current peritumoral radiomics research for NAC response assessment has primarily focused on image analysis using magnetic resonance imaging (MRI) and positron emission tomography (14–17). Considering the practical limitations that impede routine implementation of these modalities for NAC response monitoring, including substantial operational costs, extended scanning protocols, and restricted accessibility. These constraints necessitate exploration of alternative imaging approaches for longitudinal treatment assessment. While ultrasound examination has been established as a practical approach for response evaluation, conventional handheld ultrasound demonstrates limited utility in peritumoral radiomic analysis due to operator variability and inherent two-dimensional imaging constraints (18). In this context, Automated Breast Volume Scanning (ABVS) addresses these limitations through standardized image acquisition protocols (19). The volumetric capabilities of ABVS enable comprehensive visualization of tumoral and peritumoral regions, while automated acquisition eliminates operator dependence (20, 21). These advantages position ABVS as a promising platform for standardized radiomics-based analysis in routine clinical practice.

Despite the radiomics potential of ABVS technology, investigations into its application for NAC response prediction remain limited. A preliminary study by Jiang et al. (22) documented the predictive value of ABVS-based radiomics features in NAC response assessment. However, this investigation focused solely on intratumoral features, neglecting peritumoral analysis. Additionally, the study utilized conventional statistical methods rather than artificial intelligence (AI) approaches. The integration of AI-based analytical models with radiomic signatures has been demonstrated to enhance the capture of complex biological patterns and treatment outcomes, enabling objective quantification of tumor-stromal interactions that influence NAC response (23, 24).

Therefore, this study aims to identify the optimal peritumoral zone dimensions through systematic comparison of AI-driven ABVS radiomic models with varying zone parameters for NAC response prediction in BC. The established optimal parameters will be implemented in early response stratification protocols, thereby enabling evidence-based treatment modifications and minimizing patient exposure to ineffective chemotherapy regimens.

## Materials and methods

This study protocol was reviewed and approved by the Ethics Committee of The Affiliated People’s Hospital of Ningbo University (Protocol Number: 2025-007) and adhered to the principles established in the Declaration of Helsinki. Given the retrospective observational design and data analysis approach, the institutional review board determined that individual patient consent requirements could be waived. Data security protocols were established to ensure patient confidentiality through comprehensive deidentification procedures. Electronic health records were extracted and anonymized prior to analysis, with all personal identifiers removed in accordance with institutional privacy protection standards.

### Patient information

Between July 2016 and September 2024, 500 patients who received NAC for BC were retrospectively evaluated at our institution. Treatment followed standardized protocols primarily consisting of anthracycline and taxane-based NAC regimens, with additional targeted therapy and endocrine therapy administered according to molecular subtypes.

The inclusion criteria comprised: (1) histologically confirmed unifocal invasive BC without distant metastasis; (2) Eastern Cooperative Oncology Group (ECOG) performance status 0-1 (25); (3) adequate baseline organ function (hepatic, renal, hematologic, and cardiac) meeting standard chemotherapy eligibility criteria; (4) completion of standard NAC regimen; (5) pre-treatment ABVS imaging of sufficient quality for radiomics analysis; and (6) complete clinical and pathological documentation. Patients who met the inclusion criteria were subsequently excluded if they presented with: (1) significant dose modification of NAC regimen due to treatment-related toxicity; (2) tumors with excessive volume or superficial location that precluded complete delineation of the peritumoral regions; or (3) loss to follow-up before treatment completion. After applying these criteria, 402 patients were eligible for final analysis. Clinical variables including age, tumor characteristics, molecular subtypes, and treatment details were obtained from medical records.

### Treatment response assessment

The treatment protocol consisted of 6–8 cycles of NAC, followed by definitive surgery performed 2–3 weeks after chemotherapy completion. Surgical approaches were determined according to standard clinical guidelines. Tumor response was evaluated through sequential ultrasound and MRI examinations. The response classification system incorporated both pathological and radiological criteria. Patients were categorized as responders when pathological complete response was achieved, defined by the absence of invasive disease in breast tissue and lymph nodes. Response classification also included patients who exhibited minimal residual tumor cellularity or demonstrated tumor size reduction exceeding 30% based on RECIST criteria (26). Patients were classified as non-responders when disease remained stable, showed progression, or displayed tumor shrinkage below 30% from baseline measurements.

### ABVS image acquisition

Pre-treatment ABVS examinations were performed using an automated breast ultrasound system (ACUSON S2000, Siemens Medical Solutions, Mountain View, CA, USA) equipped with a mechanically-driven linear array transducer (14L5BV, 5–14 MHz). The standardized imaging protocol required patients to maintain a supine position with elevated arms and regular breathing patterns throughout the examination process. The automated mechanical arm executed systematic scanning protocols at 0.5 mm intervals to ensure complete breast volume coverage, with the transducer maintaining consistent contact pressure through automated compression. The system acquired sequential B-mode ultrasound images and reconstructed these 2D images into a comprehensive 3D volume dataset. Following data acquisition, image datasets were transferred to a dedicated post-processing workstation for automated multi-planar reconstruction, which included spatial registration of sequential frames, speckle reduction filtering, and contrast enhancement to optimize tissue differentiation (27). The protocol generated standardized axial, sagittal, and coronal views at 0.5 mm slice thickness. The reconstructed volumetric datasets were exported at native resolution (0.21 × 0.07 × 1.0 mm) with 8-bit grayscale depth for subsequent radiomics analysis.

### Image segmentation

**Figure 1:**
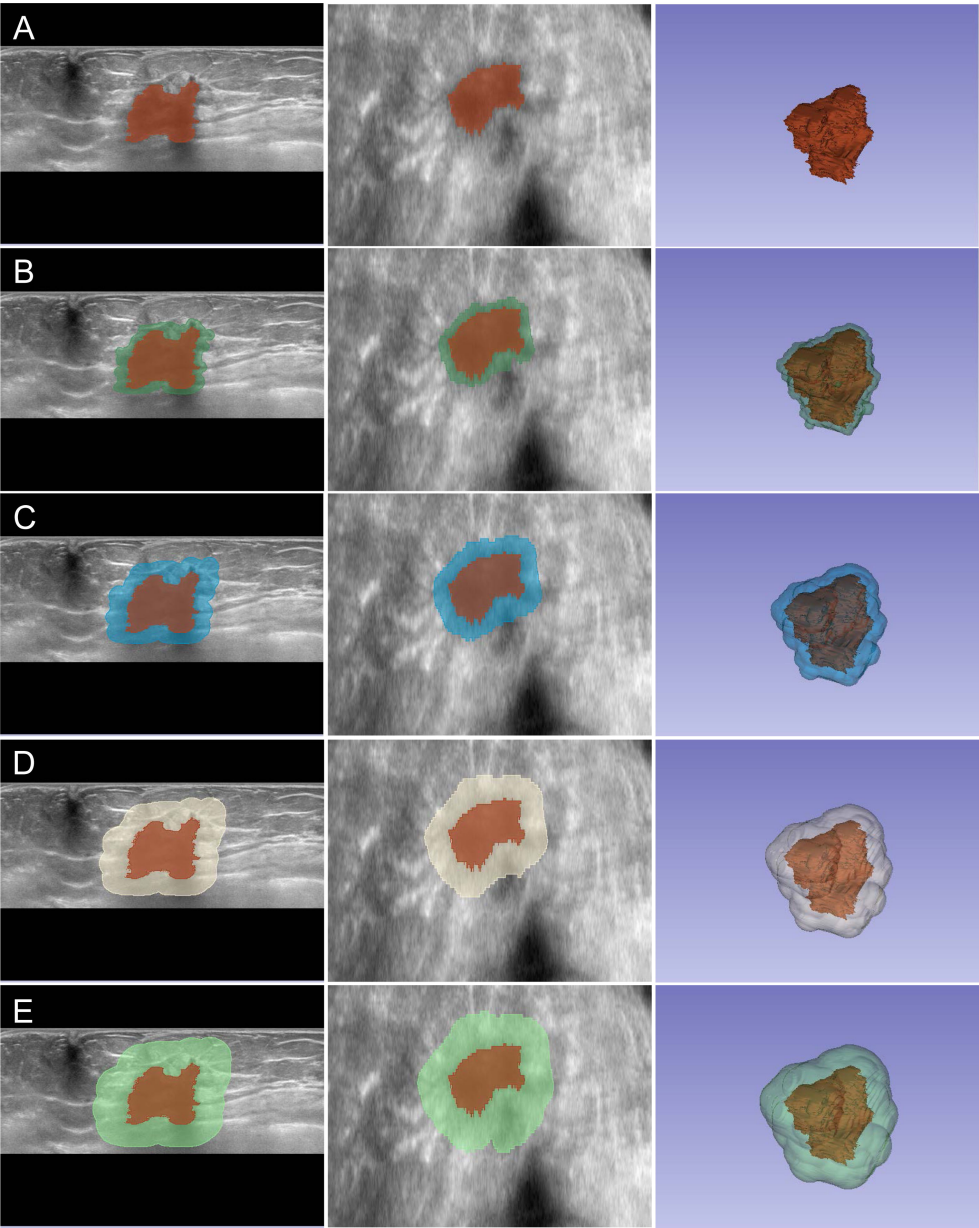
Multi-zone ROI segmentation process for radiomics analysis. Representative ABVS images showing the segmentation of intratumoral and peritumoral regions. **(A–E)** Segmentation of R0 to R8 zones displayed in axial view (left), coronal view (middle), and three-dimensional reconstruction (right). The intratumoral region (R0) is delineated in red, while peritumoral zones (R2-R8) are visualized in different colors, extending outward at 2-mm intervals from the tumor boundary.

Image analysis was performed independently by two experienced sonographers who were blinded to NAC outcomes. Tumor segmentation was conducted using 3D Slicer software (version 5.7.0) through a standardized protocol, as illustrated in **Figure 1**. Initial tumor boundaries were delineated based on grayscale differences between lesional and surrounding tissues in coronal and axial planes, with manual refinement applied to ensure accurate border definition. The analysis encompassed five distinct regions of interest (ROIs): the intratumoral zone (R0) and four peritumoral zones extending outward at 2-mm intervals (R2, R4, R6, and R8, representing regions at 2-mm, 4-mm, 6-mm, and 8-mm from tumor boundary, respectively). Prior to feature extraction, preprocessing steps included isotropic resampling (1 × 1 × 1 mm) using trilinear interpolation and gray-level normalization to 64 discrete levels. These standardization procedures minimize inter-patient acquisition variability and ensure consistent feature computation across all specimens, critical for reliable comparative radiomics analysis.

### Radiomic feature extraction

Feature extraction for radiomics analysis was performed using PyRadiomics version 3.0. The computational pipeline systematically extracted quantitative features from each defined ROI using six mathematical filters: Exponential (emphasizing high-intensity regions), Gradient (edge detection), Logarithm (dynamic range optimization), Square (intensity transformation), and Wavelet (multi-resolution decomposition). This process generated a comprehensive set of 1,409 features per ROI, encompassing shape-based descriptors, first-order statistical metrics, and textural characteristics across all filtered and original images.

### Feature preprocessing and selection

The extracted radiomic features underwent systematic preprocessing to ensure data consistency. A standardization procedure was applied wherein numerical features were subjected to Z-score normalization, followed by binary encoding for categorical parameters. Treatment outcomes were classified dichotomously, where NAC response and non-response were designated as 0 and 1, respectively. Feature refinement was executed through a hierarchical filtration protocol. The initial phase assessed feature stability between observers via Concordance Correlation Coefficient (CCC), wherein features exceeding a threshold of 0.85 were retained. These stable features were subsequently evaluated through Wilcoxon rank-sum (WRS) tests with false discovery rate adjustment (P < 0.1) to identify response-associated parameters. The final feature set was determined through minimum redundancy maximum relevance (mRMR) analysis, which yielded 30 optimal features that demonstrated significant correlation with treatment outcomes while maintaining minimal multicollinearity.

### Data splitting and model training

The study cohort was divided into training and testing sets (7:3 ratio) through stratified randomization to maintain balanced distribution of NAC response outcomes. To construct predictive models for NAC response, a TabNet deep learning framework was implemented to process the radiomic features extracted from each defined ROI, given its demonstrated capability in handling high-dimensional tabular data with inherent feature interpretation. Hyperparameter optimization for the TabNet architecture was conducted through Bayesian search methodology with 5-fold cross-validation. The optimization protocol evaluated critical parameters including decision dimension (8-64), attention dimension (8-64), number of decision steps (3-10), feature selection regularization (1.0-2.0), learning rate (1e-4 to 1e-2), and random seed (1-100). Model convergence was monitored using validation loss as the primary metric, with early stopping implemented at 10 epochs without improvement. Following optimization, the final models for each ROI were trained using 5-fold cross-validation with the identified optimal hyperparameters, and the model configuration achieving the lowest validation loss was selected for subsequent analyses.

### Model performance comparison

The ROI-specific AI models underwent systematic comparative evaluation to determine the peritumoral zone with optimal predictive value for NAC response. Model performance was evaluated in the independent testing cohort through comprehensive analysis of discrimination, calibration, and clinical utility. Performance metrics were calculated from confusion matrices according to standard classification protocols. Comparative analysis identified the model incorporating the optimal peritumoral thickness as the most robust predictor of NAC response, indicating potential applications in therapeutic decision-making protocols.

### Model interpretation

The predictive mechanism of the optimal AI model was analyzed through feature importance assessment to elucidate the underlying variables contributing to NAC response prediction. A systematic interpretation protocol was implemented to quantify the relative contribution of individual radiomic features to model predictions. The ten most influential variables were identified and visualized through feature importance analysis. An ablation study was subsequently conducted by iteratively removing the top five contributory features, followed by comparative performance evaluation to validate the significance of these identified predictive features.

### Statistical analysis

Statistical assessment protocols were implemented according to data distribution and analytical requirements. Between-group comparisons were conducted through chi-square tests for categorical variables and WRS tests for continuous variables. Model discrimination was evaluated by receiver operating characteristic (ROC) curve analysis with area under the curve (AUC) calculation. Statistical comparisons of AUC values between different models were performed using DeLong tests, with p<0.05 considered statistically significant. Model calibration was examined through calibration curves and Brier score (BS) assessment. Decision curve analysis (DCA) was performed to evaluate clinical utility through net benefit calculation across probability thresholds. Performance metrics were derived from confusion matrices, including accuracy, precision, recall, F1-score and Kappa values. All statistical analyses were performed using Python version 3.12.0 with established statistical libraries.

## Results

### Patient characteristics and treatment outcomes

**Figure 2:**
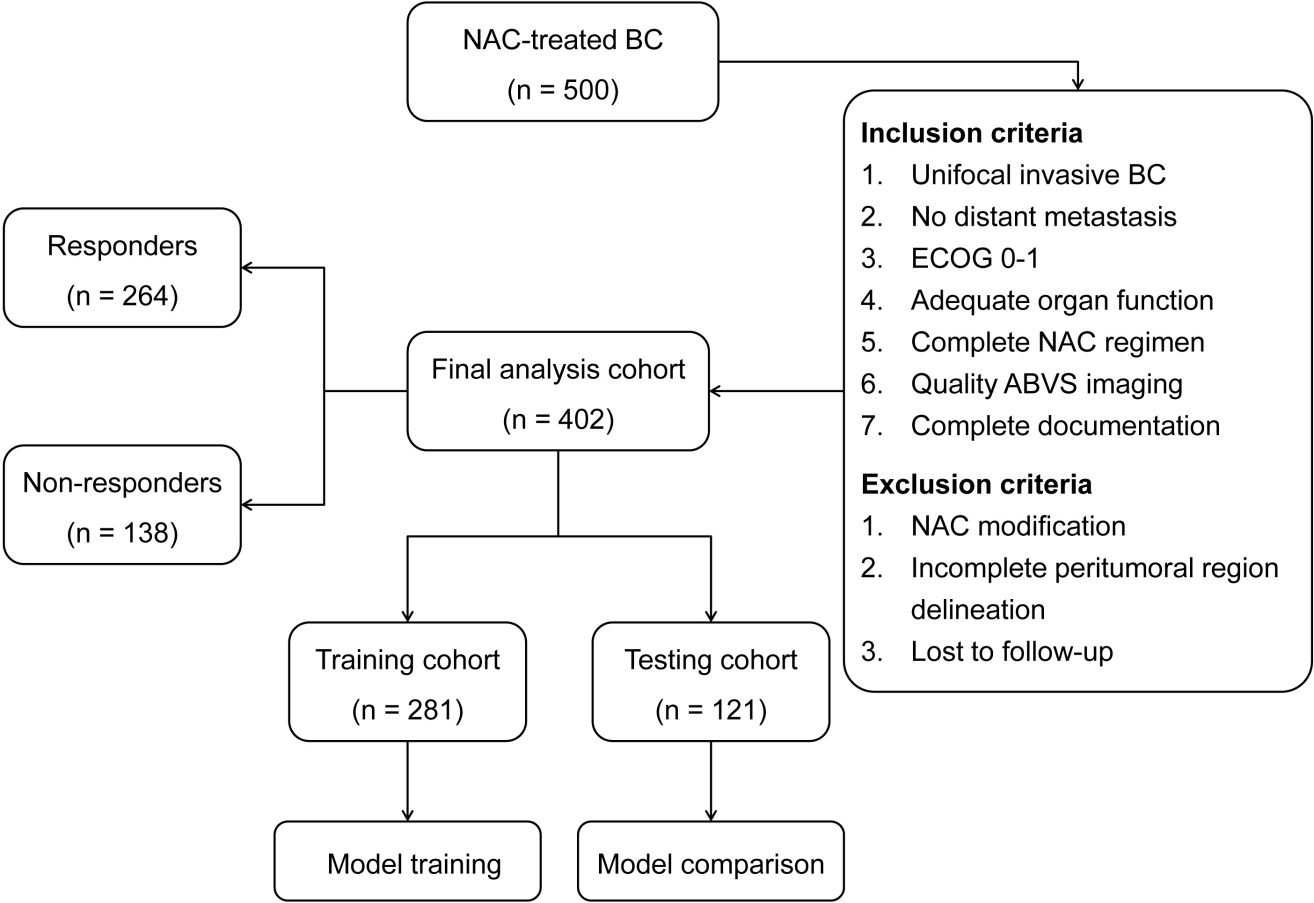
Study flowchart illustrating patient selection and cohort distribution.

The final study population included 402 BC patients who met the selection criteria. The neoadjuvant chemotherapy protocols consisted of AC-T (doxorubicin, cyclophosphamide, followed by paclitaxel) in 254 patients (63.2%) and FEC-D (fluorouracil, epirubicin, cyclophosphamide, followed by docetaxel) in 117 patients (29.1%). All patients received standard doses with a median of 6 cycles (range: 6-8). Trastuzumab was administered concurrently to HER2-positive patients (n=58, 14.4%). Based on established response criteria, 264 patients (65.7%) were classified as responders and 138 (34.3%) as non-responders, with no significant differences in demographic or clinical characteristics between response groups (P > 0.05). The cohort was divided into training (n=281, 69.9%) and testing (n=121, 30.1%) sets, comprising 97 (34.5%) and 41 (33.9%) non-responders, respectively. The patient selection process and cohort distribution are presented in **Figure 2**. No significant differences were observed in demographic and clinical characteristics between training and testing cohorts (P > 0.05), as summarized in **Table 1**.

**Table 1:** Patient and tumor characteristics.

Characteristics		Training cohort (n=281)			Testing cohort (n=121)		
		Response (n=184)	Non-response (n=97)	P value	Response (n=80)	Non-response (n=41)	P value
Age, years		50 (48, 53)	52 (47, 56)	0.384	51 (48,54)	54 (46,59)	0.274
Menopausal status, n(%)	Premenopausal	103 (56.0%)	57 (58.8%)	0.654	47 (58.8%)	26 (63.4%)	0.620
	Postmenopausal	81 (44.0%)	40 (41.2%)		33 (41.3%)	15 (36.6%)	
Initial tumor size, cm		4.6 (3.7, 5.4)	4.8 (3.9, 5.7)	0.482	4.5 (3.5,5.3)	4.8 (3.7,5.8)	0.251
Clinical stage, n(%)	Stage II	58 (31.5%)	24 (24.7%)	0.235	27 (33.8%)	12 (29.3%)	0.618
	Stage III	126 (68.5%)	73 (75.3%)		53 (66.2%)	29 (70.7%)	
Molecular subtype, n(%)	HR+/HER2−	62 (33.7%)	40 (41.2%)	0.351	27 (33.8%)	13 (31.7%)	0.193
	HER2+	96 (52.2%)	42 (43.3%)		45 (56.2%)	19 (46.3%)	
	TNBC	26 (14.1%)	15 (15.5%)		8 (10.0%)	9 (22.0%)	
Histological type, n(%)	IDC	140 (76.1%)	81 (83.5%)	0.239	67 (83.8%)	38 (92.7%)	0.327
	ILC	33 (17.9%)	10 (10.3%)		11 (13.7%)	2 (4.9%)	
	Other	11 (6.0%)	6 (6.2%)		2 (2.5%)	1 (2.4%)	
Chemotherapy regimen, n(%)	AC-T	110 (59.8%)	62 (63.9%)	0.335	57 (71.2%)	25 (61.0%)	0.133
	FEC-D	55 (29.9%)	30 (30.9%)		17 (21.3%)	15 (36.6%)	
	Others	19 (10.3%)	5 (5.2%)		6 (7.5%)	1 (2.4%)	
Surgery type, n(%)	Mastectomy	129 (70.1%)	75 (77.3%)	0.198	63 (78.8%)	30 (73.2%)	0.491
	Breast-conserving	55 (29.9%)	22 (22.7%)		17 (21.3%)	11 (26.8%)	

Quantitative data are presented as median (interquartile range). Categorical data are presented as number (percentage). HR, hormone receptor, HER2, human epidermal growth factor receptor-2, TNBC, triple-negative breast cancer, IDC, Invasive ductal carcinoma, ILC, Invasive lobular carcinoma

### Radiomics feature extraction and selection

The quantitative image analysis yielded 7,045 radiomic features per patient (1,409 features × 5 ROIs) from the pre-NAC ABVS images. Following standardization, feature reproducibility assessment identified stable features (CCC ≥ 0.85) for subsequent analysis, retaining 77.4%, 84.1%, 82.8%, 76.5%, and 78.8% of features from R0 to R8, respectively. Statistical comparison between responder and non-responder groups identified discriminative features (163, 182, 154, 172, and 147 features from R0 to R8, respectively). Through mRMR algorithm optimization, 30 features were selected from each ROI, encompassing first-order statistics, shape-based descriptors, and various texture parameters. The detailed distribution of these selected features across different categories and ROIs is presented in **Supplementary Table S1**. Multiple mathematical filters, including wavelet, logarithm, exponential, and gradient transformations, were applied during feature extraction to enhance the characterization of tissue heterogeneity in both intratumoral and peritumoral regions.

### Model training and optimization

Independent TabNet models were developed for each ROI using the training cohort data to predict NAC response. Through Bayesian optimization with 5-fold cross-validation, region-specific hyperparameter configurations were established for the five distinct models (R0-R8). The optimal hyperparameter settings for each model are presented in **Supplementary Table S2**. Model training was executed using these optimized configurations, with the best-performing model iteration selected based on minimal validation loss criteria. The model training process is illustrated in **Supplementary Figure S1**, depicting the convergence of loss functions across epochs, while **Supplementary Figure S2** demonstrates the corresponding accuracy trajectories for both training and validation sets.

### Model performance comparison

**Figure 3:**
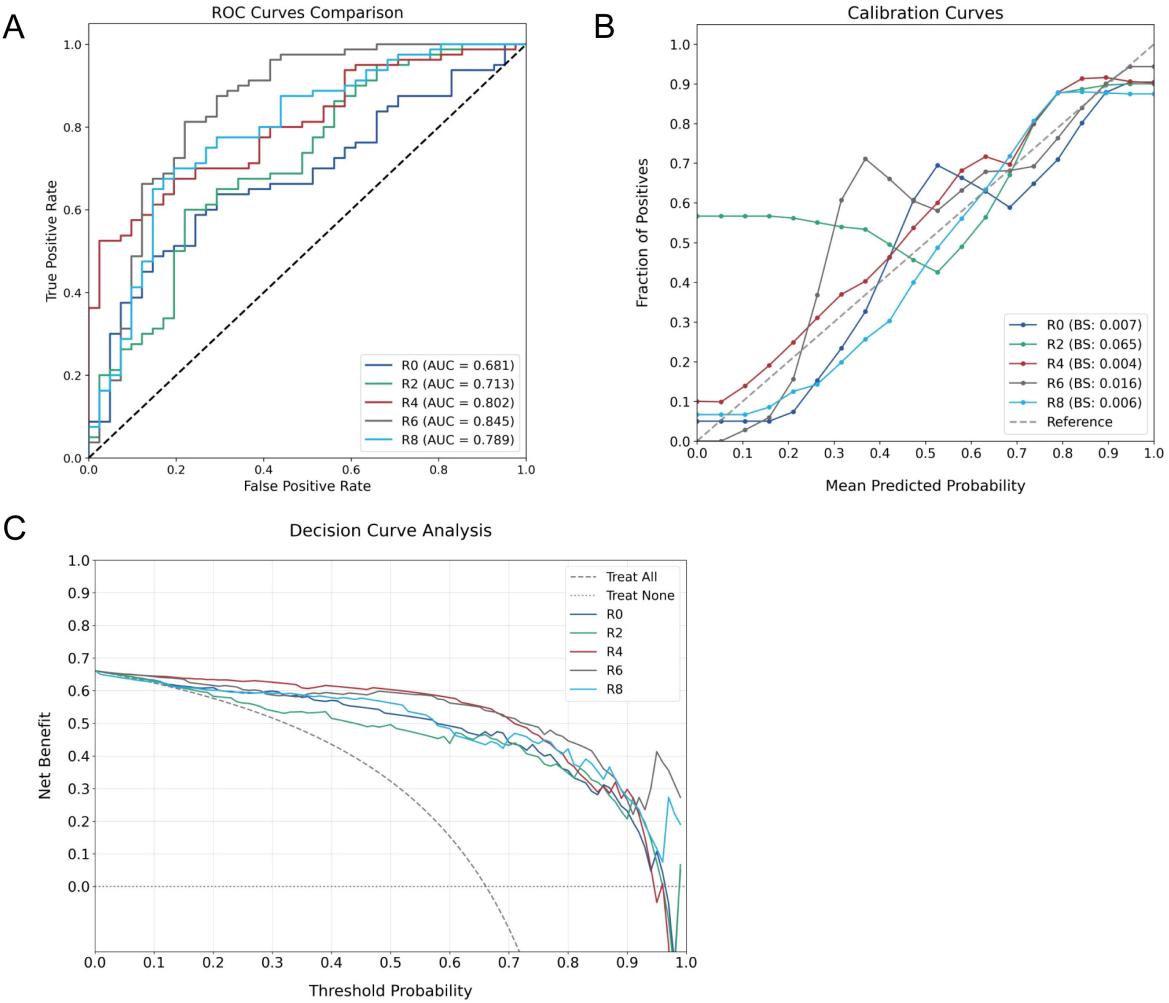
Comprehensive performance evaluation of ROI-specific models. **(A)** ROC curves comparing discriminative capabilities across models. **(B)** Calibration plots assessing probability estimation accuracy. **(C)** DCA demonstrating clinical utility across different threshold probabilities.

The predictive performance of ROI-specific models was systematically evaluated in the testing cohort. Discrimination analysis through ROC curves demonstrated a progressive improvement in predictive accuracy from intratumoral to peritumoral regions, with AUC values increasing from 0.681 (R0) to 0.845 (R6), followed by a decline in R8 (0.789) (**Figure 3A**). DeLong tests revealed that the R6 model demonstrated statistically significant improvement compared to R0 and R2 models, with pairwise comparisons of AUC values presented in **Table 2**. Though differences between R6 and R4 or R8 did not reach statistical significance, R6 consistently maintained the highest numerical AUC value across all models.

**Table 2:** Pairwise comparisons of AUC values between ROI-specific models using DeLong test.

Model 1 vs Model 2	AUC difference	P-value
R0 vs R2	0.032	0.750
R0 vs R4	0.122	0.204
R0 vs R6	0.216	0.013
R0 vs R8	0.108	0.263
R2 vs R4	0.089	0.343
R2 vs R6	0.183	0.032
R2 vs R8	0.076	0.425
R4 vs R6	0.094	0.231
R4 vs R8	0.013	0.880
R6 vs R8	0.107	0.177

The calibration assessment demonstrated varying reliability patterns across models, with BS values consistently below 0.1. Notably, R2 exhibited suboptimal calibration performance, while R6 showed characteristic deviation in the low-probability range (0.3-0.4), where the observed non-responder rate (0.6-0.7) exceeded model predictions, indicating potential underestimation of non-responder probability (**Figure 3B**). DCA indicated sustained clinical utility across varying threshold probabilities for all models (**Figure 3C**).

Detailed performance metrics derived from confusion matrices (**Table 3**) demonstrated superior classification in peritumoral models R2-R6. Through comprehensive evaluation of model performance, R6 emerged as the optimal predictor, achieving the highest precision (0.765) and accuracy (0.810). While this model demonstrated relatively lower recall (0.634) due to increased false negatives, its superior precision in identifying non-responders offers substantial clinical value. This characteristic is particularly beneficial for treatment planning, as accurate identification of NAC non-responders enables timely therapeutic strategy modification, potentially preventing unnecessary treatment exposure.

**Table 3:** Detailed performance metrics of ROI-specific models in the testing cohort.

Model	Accuracy	Precision	Recall	F1 score	Kappa	TP	TN	FP	FN
R0	0.661	0.500	0.780	0.610	0.335	32	48	32	9
R2	0.802	0.689	0.756	0.721	0.568	31	66	14	10
R4	0.802	0.730	0.659	0.692	0.547	27	70	10	14
R6	0.810	0.765	0.634	0.693	0.557	26	72	8	15
R8	0.711	0.552	0.780	0.646	0.414	32	54	26	9

TP, true positive; TN, true negative; FP, false positive; FN, false negative.

### Combined model performance

Following the identification of R6 as the optimal peritumoral zone for NAC response prediction, we developed a combined model integrating radiomic features from both R0 and R6 to leverage the complementary information provided by these distinct tumor-associated regions. The combined R0+R6 model demonstrated enhanced predictive capability compared to individual regional models. By leveraging the high recall of the R0 model in conjunction with the enhanced precision of the R6 model, the integrated approach achieved a notable reduction in false negative predictions, with only 4 cases misclassified compared to 15 in the R6 model. Quantitative evaluation revealed superior performance across all metrics, with accuracy of 0.909, precision of 0.841, recall of 0.902, and F1 score of 0.871. The model achieved a robust Kappa of 0.801, indicating substantial agreement beyond chance.

### Key feature identification

**Figure 4:**
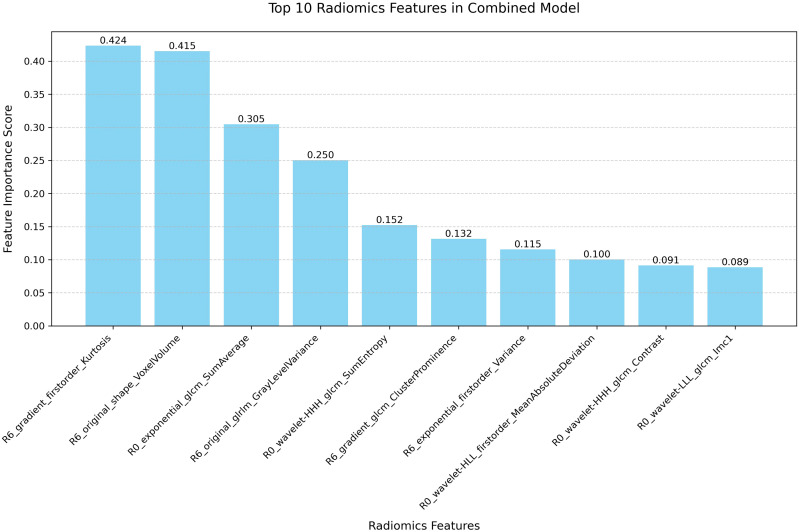
Feature importance ranking of the combined model. Bar plot illustrating the relative contribution of the top 10 radiomic features in the combined R0 and R6 model.

Feature importance analysis was performed through the intrinsic attribution mechanism of TabNet to identify crucial predictive variables in the combined model (**Figure 4**). Among the top ten contributory features, gradient-based first-order statistics and morphological parameters demonstrated predominant influence in NAC response prediction. R6_gradient_firstorder_Kurtosis and R6_original_shape_VoxelVolume were identified as the most significant predictors, highlighting the importance of peritumoral textural heterogeneity and volumetric characteristics. The impact of these features was validated through systematic ablation analysis (**Table 4**). Sequential elimination of the top five variables revealed progressive performance deterioration, with removal of R6_gradient_firstorder_Kurtosis and R6_original_shape_VoxelVolume leading to substantial precision reduction. These findings indicate the fundamental role of peritumoral textural and morphological features in characterizing tumor-stromal interactions associated with treatment response.

**Table 4:** Ablation analysis of top five features in the combined model.

Model Configuration	Accuracy	Precision	Recall	F1 score	Kappa	TP	TN	FP	FN
Combined Model	0.909	0.841	0.902	0.871	0.801	37	73	7	4
Without R6_gradient_firstorder_Kurtosis	0.769	0.638	0.732	0.682	0.440	30	63	17	11
Without R6_original_shape_VoxelVolume	0.818	0.721	0.756	0.738	0.497	31	68	12	10
Without R0_exponential_glcm_SumAverage	0.851	0.780	0.780	0.780	0.668	32	71	9	9
Without R6_original_glrlm_GrayLevelVariance	0.860	0.816	0.756	0.785	0.681	31	73	7	10
Without R0_wavelet-HHH_glcm_SumEntropy	0.876	0.842	0.780	0.810	0.718	32	74	6	9

TP, true positive; TN, true negative; FP, false positive; FN, false negative.

## Discussion

Accurate prediction of NAC response remains crucial for BC management, as early identification of potential non-responders enables timely therapeutic modifications and prevents unnecessary treatment exposure. While peritumoral radiomics analysis offers a non-invasive approach for treatment response assessment, systematic evaluation of optimal peritumoral dimensions for predictive accuracy remains unexplored in current literature. This methodological limitation undermines the potential of peritumoral radiomics in characterizing tumor-stromal interactions that influence treatment outcomes. In this study, multiple AI-driven radiomics models were developed and validated using ABVS images across varying peritumoral thicknesses. The findings demonstrated superior predictive performance through integration of intratumoral features with those from the 6-mm peritumoral region, achieving an accuracy of 0.909 and precision of 0.841 in the testing cohort. This standardized ultrasound-based approach enables broad clinical implementation, facilitating more personalized therapeutic decision-making.

The superior predictive capability of peritumoral regions in NAC response assessment can be attributed to the complex tumor microenvironment, which comprises immune cells, stromal cells, and extracellular matrix components that collectively influence therapeutic outcomes (28, 29). Our systematic analysis revealed that predictive accuracy progressively improved from intratumoral to 6-mm peritumoral regions before declining at 8-mm, suggesting an optimal zone for capturing tumor-stromal interactions. This spatial pattern aligns with biological evidence demonstrating that the immediate peritumoral environment exhibits higher immune cell density and more active tumor-stromal crosstalk compared to distant regions (30). Particularly, tumor-infiltrating lymphocyte levels within the peritumoral microenvironment have been significantly correlated with pathological complete response to NAC (31, 32). The 6-mm peritumoral zone likely represents a critical threshold where the radiomics features optimally capture these biological interactions while minimizing the inclusion of non-specific tissue characteristics. This observation is supported by previous studies showing that peritumoral features within defined margins provide more accurate characterization of tumor biology and treatment response (14, 33). The observed decline in predictive performance beyond 6-mm may reflect the diminishing influence of tumor-associated molecular and cellular alterations at greater distances from the tumor boundary. This aligns with evidence that the immediate peritumoral environment exhibits stronger biological interactions compared to more distant regions (12, 34, 35).

In this study, the TabNet architecture was selected for ABVS radiomics analysis based on demonstrated capabilities in processing high-dimensional tabular data and providing interpretable features. The architecture incorporates sequential attention mechanisms and feature selection processes that are particularly effective for handling imbalanced datasets commonly encountered in medical prediction tasks. These mechanisms enable differential weighting of features through attention layers, which inherently mitigate bias toward majority classes often observed with conventional algorithms. This characteristic allows robust performance to be achieved without requiring artificial data manipulation techniques such as oversampling (36). Through comprehensive model comparison, the 6-mm peritumoral zone exhibited optimal predictive performance, achieving the highest accuracy through superior precision values. The enhanced precision in identifying NAC non-responders offers substantial clinical value by enabling confident early identification of patients requiring alternative therapeutic strategies. Although the R6 model showed relatively lower recall values, we observed that the R0 model demonstrated higher recall capability, providing a complementary foundation for model integration. This complementarity likely stems from the distinct biological information captured in each region. Intratumoral features may primarily reflect inherent tumor properties such as cellular density and necrotic patterns that are sensitive to various resistance mechanisms, potentially capturing more cases but with lower specificity. Peritumoral features likely represent tumor-stromal interactions and immune infiltration patterns that, when detected, more reliably indicate established resistance mechanisms, offering greater precision in predictions. By leveraging these complementary characteristics, the combined model demonstrated superior performance across all evaluation metrics, substantially reducing false negatives from 15 to 4 cases and establishing a robust framework for ABVS-based NAC response prediction.

Feature importance analysis of the combined model revealed distinct patterns in the contribution of various mathematical filters to predictive radiomic features. Wavelet transformations emerged as the most significant contributors, accounting for 4 of the top 10 features, with particular effectiveness in characterizing intratumoral heterogeneity through multi-resolution decomposition. Gradient-based filters, which specifically enhance edge detection, demonstrated superior performance in quantifying peritumoral textural properties, suggesting their value in characterizing the tumor-stroma interface. Exponential filters, designed to emphasize high-intensity regions, contributed proportionally to both intratumoral and peritumoral feature extraction. This differential pattern of filter contribution indicates that complex tissue interfaces and heterogeneity patterns are optimally captured through complementary mathematical transformations. Notably, original (unfiltered) features maintained significant predictive value, particularly for structural and volumetric assessment in the peritumoral environment, highlighting the importance of preserving native image characteristics alongside enhanced feature extraction.

Among the top ten features, peritumoral characteristics demonstrated predominant influence, with R6_gradient_firstorder_Kurtosis and R6_original_shape_VoxelVolume ranking as the most significant predictors. The gradient-enhanced firstorder_Kurtosis reflects the heterogeneity of tissue density distributions within the peritumoral region, potentially capturing the complex immunological and stromal interactions that influence treatment response (37). Recent studies have demonstrated that kurtosis specifically correlates with tumor-infiltrating lymphocyte density and distribution patterns in BC, where higher kurtosis values indicate more heterogeneous immune infiltration associated with improved therapeutic response (2, 38). The volumetric characteristics derived from shape-based features indicate the spatial extent of tumor-associated alterations in the surrounding tissue architecture. Research has shown that these volumetric parameters correlate with extracellular matrix remodeling and vascular proliferation intensity in the peritumoral environment (39, 40), which directly influences drug delivery efficiency and subsequent treatment outcomes. Ablation analysis validated the critical role of peritumoral features, as removal of gradient-based textural features and volumetric characteristics substantially impacted model performance, reducing precision from 0.841 to 0.638 and 0.721, respectively. These findings demonstrate that peritumoral textural heterogeneity and morphological features provide essential information for treatment response prediction, likely reflecting underlying biological processes such as inflammatory responses and stromal remodeling that influence NAC outcomes.

The developed combined model demonstrates clinical utility through ABVS-based radiomics analysis of intratumoral and peritumoral regions for NAC treatment personalization. Early identification of potential non-responders enables consideration of alternative therapeutic protocols, including chemotherapy regimen modifications or immediate surgical intervention, thereby preventing unnecessary treatment exposure and optimizing clinical outcomes. However, several limitations warrant consideration in our study. The single-center retrospective design with limited sample size introduces potential overfitting risk and necessitates validation through multi-institutional prospective investigations. Our methodological approach required patients with histologically confirmed unifocal BC without distant metastasis and full delineation of peritumoral regions, necessitating the exclusion of excessively large tumors and those with superficial locations. The application of these strict selection criteria might have resulted in a cohort with notably homogeneous clinical characteristics, as evidenced by the absence of significant differences between response groups. This homogeneity introduces a potential selection bias that limits the generalizability of our findings, particularly to patient populations with larger tumor burdens or multifocal disease presentations. Additionally, our analysis was confined to pre-treatment ABVS examinations, whereas longitudinal imaging during NAC cycles might provide valuable temporal predictive indicators for treatment response (22). The absence of direct pathological correlation with specific peritumoral regions introduces potential validation bias, although systematic pathological sampling presents practical and ethical limitations. Future investigations should address prospective validation, external dataset testing, temporal imaging feature integration, and model development for diverse tumor presentations. Integration of molecular and pathological markers with radiomics features may enhance biological interpretation of peritumoral signatures and improve prediction accuracy.

## Conclusion

In conclusion, this study established a systematic framework utilizing TabNet deep learning architecture for determining optimal peritumoral thickness in ABVS-based radiomics analysis for NAC response prediction in BC. The 6-mm peritumoral zone demonstrated superior predictive capability, with the AI-driven combined intratumoral-peritumoral radiomics model achieving optimal performance through enhanced accuracy and precision. This standardized approach enables robust pre-treatment response assessment through readily accessible ultrasound technology, potentially facilitating early identification of NAC non-responders and supporting personalized therapeutic decision-making in clinical practice.

## Data Availability

The original contributions presented in the study are included in the article/**Supplementary Material**. Further inquiries can be directed to the corresponding authors.
