# Supplementary material for: Comparative analysis of multi-zone peritumoral radiomics in breast cancer for predicting NAC response using ABVS-based deep learning models
Source: Front Oncol. 2025 May 14;15:1586715. doi: 10.3389/fonc.2025.1586715 (PMC12116539; doi:10.3389/fonc.2025.1586715)
Supplement: Supplementary file 1 [file DataSheet1.docx]

**Supplementary**

**Table S1**. Distribution of selected radiomic features across ROIs

| Feature Category | | R0 | R2 | R4 | R6 | R8 |
| --- | --- | --- | --- | --- | --- | --- |
| First-order Statistics | | 8 | 7 | 8 | 9 | 8 |
| Shape-based | | 2 | 1 | 1 | 1 | 0 |
| Texture Features | GLCM | 7 | 8 | 7 | 7 | 8 |
|  | GLRLM | 5 | 4 | 4 | 4 | 4 |
|  | GLSZM | 4 | 5 | 5 | 4 | 5 |
|  | GLDM | 3 | 4 | 4 | 4 | 4 |
|  | NGTDM | 1 | 1 | 1 | 1 | 1 |

GLCM: Gray Level Co-occurrence Matrix, GLRLM: Gray Level Run Length Matrix, GLSZM: Gray Level Size Zone Matrix, GLDM: Gray Level Dependence Matrix, NGTDM: Neighborhood Gray Tone Difference Matrix

**Table S2**. Optimal hyperparameters for TabNet models across different ROIs

| Hyperparameter | R0 | R2 | R4 | R6 | R8 |
| --- | --- | --- | --- | --- | --- |
| Decision dimension | 18 | 32 | 16 | 32 | 16 |
| Attention dimension | 32 | 20 | 16 | 32 | 16 |
| Decision steps | 5 | 4 | 3 | 5 | 4 |
| Feature selection regularization | 1.5 | 1.5 | 1.2 | 1.5 | 1.2 |
| Learning rate | 0.001 | 0.001 | 0.01 | 0.01 | 0.001 |
| Random seed | 123 | 256 | 42 | 123 | 42 |


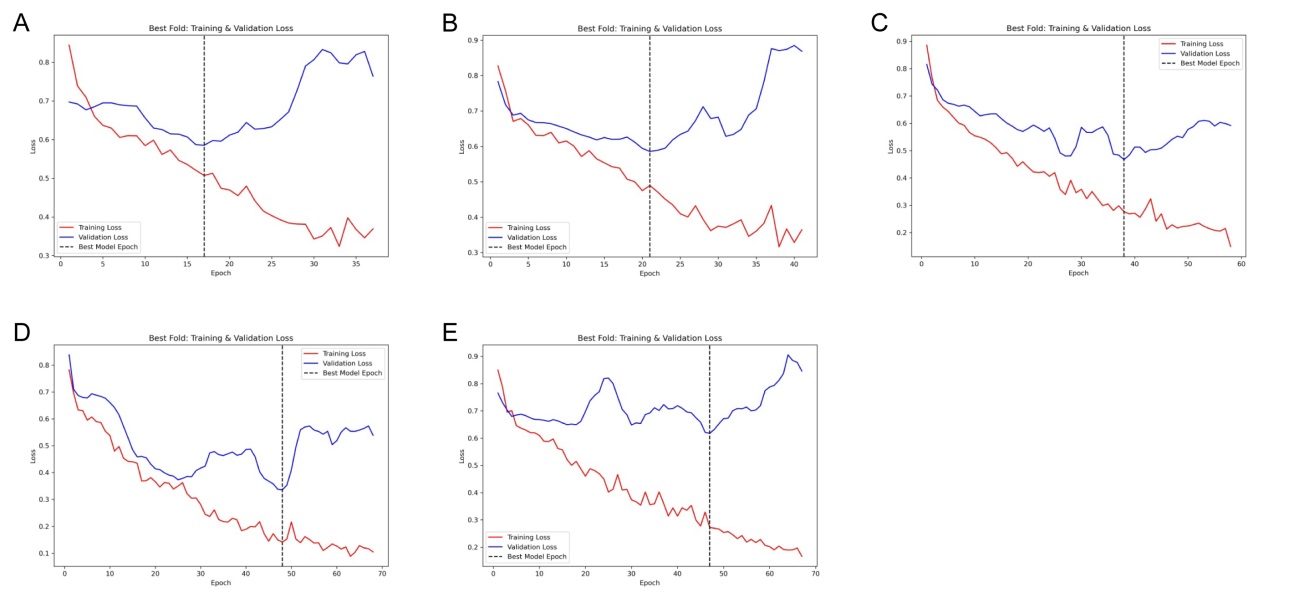


**Figure S1.** Training and validation loss curves across different ROI models. Loss trajectories for TabNet models trained on (A) intratumoral region (R0), (B-E) peritumoral regions R2-R8, demonstrating model convergence patterns during the training process. The vertical dotted line indicates the epoch at which the optimal model was selected based on minimal validation loss.


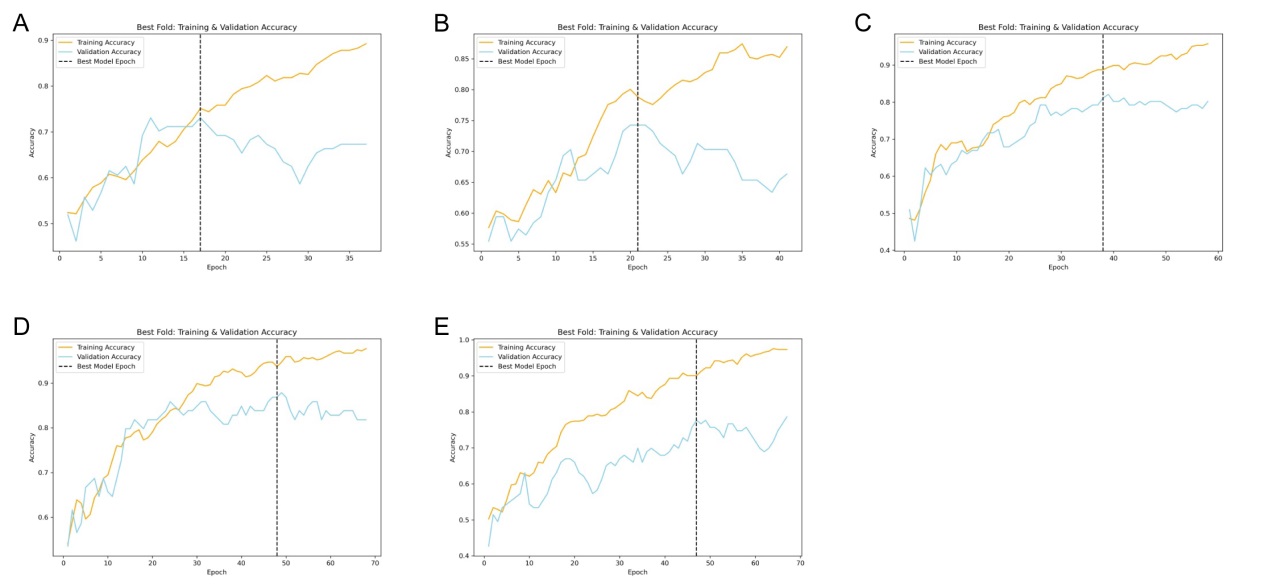


**Figure S2**. Training and validation accuracy curves across different ROI models. Accuracy trajectories for TabNet models trained on (A) intratumoral region (R0), (B-E) peritumoral regions R2-R8, illustrating the classification performance evolution during the training process. The vertical dotted line indicates the epoch corresponding to the optimal model selection based on validation loss criteria.
